# Roux-en-Y Gastric Bypass Improves Adiponectin to Leptin Ratio and Inflammatory Profile in Severely Obese Women with and without Metabolic Syndrome: A Randomized Controlled Trial

**DOI:** 10.3390/nu15153312

**Published:** 2023-07-26

**Authors:** Sandra M. B. P. Moreira, André L. L. Bachi, Elias I. Jirjos, Carlos A. Malheiros, Sergio Vencio, Vera L. S. Alves, Alan R. T. Sousa, Lucenda A. Felipe, Eduardo A. Perez, Maria E. M. Lino, Shayra K. A. Souza, Juliana M. B. Santos, Miriã C. Oliveira, Adriano L. Fonseca, Carlos H. M. Silva, Rodolfo P. Vieira, Giuseppe Insalaco, Wilson R. Freitas Júnior, Luis V. F. Oliveira

**Affiliations:** 1 Post Graduation Program in Health Sciences, Santa Casa of São Paulo Medical School, São Paulo 01224-001, SP, Brazil; sandra.moreira@goias.gov.br (S.M.B.P.M.); eliasjilias@gmail.com (E.I.J.); camalheiros@gmail.com (C.A.M.); fisioterapiasc@uol.com.br (V.L.S.A.); alanrts@gmail.com (A.R.T.S.); lucendafelipe1977@gmail.com (L.A.F.); dudu.perez@hotmail.com (E.A.P.); wilsonrfreitasjunior@gmail.com (W.R.F.J.); 2 Post-Graduation Program in Health Sciences, Santo Amaro University (UNISA), São Paulo 04829-300, SP, Brazil; allbachi77@gmail.com; 3 Institute of Pharmaceutical Sciences, Goiania 74175-100, GO, Brazil; svencio@gmail.com; 4 Scientific Initiation Program, Evangelical University of Goiás (UniEVANGELICA), Anápolis 75083-515, GO, Brazil; maria.eduardamlino@hotmail.com (M.E.M.L.); shayra.kas@gmail.com (S.K.A.S.); 5 Department of Physical Therapy, School of Medicine, University of São Paulo, São Paulo 05360-000, SP, Brazil; juliana-mbs@hotmail.com; 6 Human Movement and Rehabilitation Post Graduation Program, Evangelical University of Goiás (UniEVANGELICA), Anápolis 75083-515, GO, Brazil; miriacandidaoliveira@gmail.com (M.C.O.); fonseca.luis.adriano@gmail.com (A.L.F.); carloshmendes@unievangelica.edu.br (C.H.M.S.); rodrelena@yahoo.com.br (R.P.V.); 7 Institute of Translational Pharmacology, National Research Council of Italy (CNR), 90146 Palermo, Italy; giuseppe.insalaco@ift.cnr.it

**Keywords:** Roux-en-Y gastric bypass, adiponectin to leptin ratio, inflammatory profile, severely obese, metabolic syndrome

## Abstract

Obesity is a troubling public health problem as it increases risks of sleep disorders, respiratory complications, systemic arterial hypertension, cardiovascular diseases, type 2 diabetes mellitus, and metabolic syndrome (MetS). As a measure to counteract comorbidities associated with severe obesity, bariatric surgery stands out. This study aimed to investigate the adiponectin/leptin ratio in women with severe obesity with and without MetS who had undergone Roux-en-Y gastric bypass (RYGB) and to characterize the biochemical, glucose, and inflammatory parameters of blood in women with severe obesity before and after RYGB. Were enrolled females with severe obesity undergoing RYGP with MetS (*n* = 11) and without (*n* = 39). Anthropometric data and circulating levels of glucose, total cholesterol, high-density lipoprotein (HDL), non-HDL total cholesterol, low-density lipoprotein (LDL), adiponectin, and leptin were assessed before and 6 months after RYGB. Significant reductions in weight, body mass index, and glucose, total cholesterol, LDL, and leptin were observed after surgery, with higher levels of HDL, adiponectin, and adiponectin/leptin ratio being observed after surgery compared to the preoperative values of those. This study demonstrated that weight loss induced by RYGB in patients with severe obesity with or without MetS improved biochemical and systemic inflammatory parameters, particularly the adiponectin/leptin ratio.

## 1. Introduction

The rise in obesity in recent years has been alarming, and it has become a serious public health concern worldwide. According to the World Health Organization (WHO), 39% of adults over 18 years of age in 2016 were overweight and 13% were obese [1]. As the conditions of being overweight and obesity increase the risk of chronic and aggressive conditions such as respiratory complications, systemic arterial hypertension (SAH), type 2 diabetes mellitus (T2DM), sleep disorders, cancer, and cardiovascular diseases, these numbers are concerning [2].

Particularly for the cardiovascular system, obesity initiates both direct effects via structural and functional adaptations owing to excess body weight, and indirect effects through adipokines that induce a pro-inflammatory and pro-thrombotic state mediated by insulin resistance, T2DM, visceral obesity, SAH, and dyslipidemia [3,4,5]. When indirect effects occur in the same patient, it is labeled metabolic syndrome (MetS), which, according to the National Cholesterol Education Program (NCEP), occurs when the simultaneous presence of hyperglycemia/insulin resistance, visceral obesity, dyslipidemia, and SAH is observed [6,7,8]. The prevalence of MetS varies from 20 to 25% in the adult population [9,10] and from 0 to 19.2% [11] in children, and has the potential of reaching 80% in patients with T2DM [12]. Although it has been accepted that obesity leads to the establishment of systemic inflammatory conditions and is closely associated with MetS, the pathophysiology of MetS is still not well-established, with some studies suggesting inflammation as an etiology [13,14].

It is noteworthy that in obesity, the accumulation of fat in adipocytes plays a marked role in the inflammatory process; as the volume of adipose tissue increases, so does the production of adipocytokines, triggering diverse pathophysiological processes that are inflammation-related. [15]. This long-term clinical picture of low-grade inflammation is an important risk factor for the emergence of some types of cancer, non-alcoholic fatty liver disease, atherosclerosis, SAH, T2DM, and MetS [16].

Among the various biomarkers related to obesity and MetS, adiponectin, leptin, C-reactive protein (CRP), and cytokines such as interleukins (ILs) -1 and -6 stand out (13). Interestingly, according to the global scientific literature, the increase in circulating concentrations of leptin in contrast to the decrease in adiponectin levels is a striking aspect of both obesity and MetS. Thus, the adiponectin/leptin ratio can be considered a biomarker of inflammation in adipose tissue [17]. 

Based on this information, it is evident that the management of obesity and MetS through lifestyle changes, regular physical activity, weight loss pharmacotherapy, the use of gastric devices, and bariatric surgeries is essential to minimize the deleterious effects of these conditions [18]. In particular, bariatric surgery (BS) is an effective alternative for reducing excess body weight and leads to a reduction in cardiovascular risk, dyslipidemia, non-alcoholic fatty liver disease, insulin resistance, and T2DM among other obesity-related metabolic conditions. BS is also closely associated with not only a significant decrease in systemic levels of leptin but also an increase in adiponectin levels, as it encourages satiety via hormonal regulation and a reduction in stomach volume [19,20,21,22,23,24]. It is noteworthy that patients undergoing BS must also receive adequate nutritional and psychological monitoring to achieve the goals of reducing their body mass index (BMI) and maintaining a healthy metabolic state [25]. The appropriate surgical procedure should be indicated according to the individual factors and comorbidities of each patient as well as the confidence and experience of the surgeon [26].

It has been reported that patients with severe obesity, with or without T2DM, present with significant and sustained weight loss with a consequent reduction in several mediators, some inflammatory, with elevations in adiponectin values in association with a decrease in leptin and tumor necrosis factor-alpha (TNF-α) levels after BS through Roux-en-Y gastric bypass (RYGB) [27,28]. Given this background, this study aimed to investigate the adiponectin/leptin ratio in women with severe obesity with and without MetS before and after RYGB and to characterize the biochemical markers, such as circulating levels of glucose, total cholesterol, high-density lipoprotein (HDL), non-HDL total cholesterol, low-density lipoprotein (LDL), adiponectin, and leptin.

## 2. Materials and Methods

### 2.1. Trial Design

**Figure 1:**
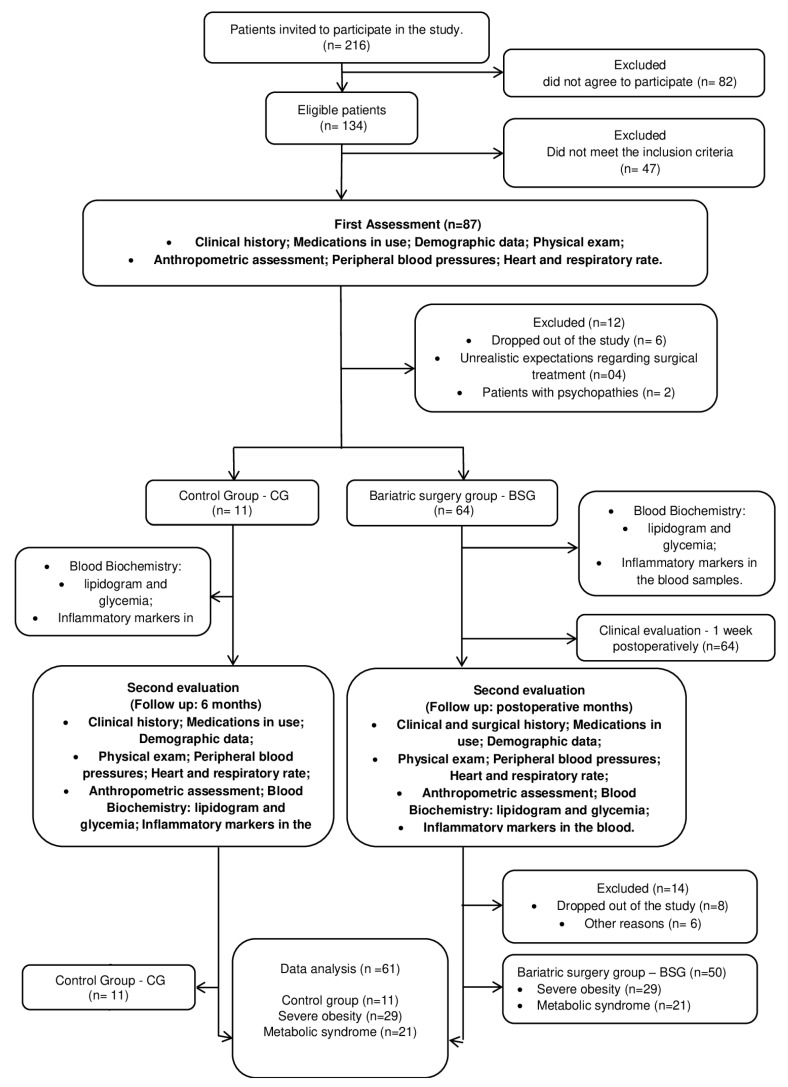
Study flowchart.

In the present randomized controlled clinical trial, female patients were enrolled who presented a clinical diagnosis of severe obesity with or without MetS, and also who were indicated for bariatric surgery. To perform this study, the Standard Protocol Items: Recommendations for Interventional Trials (SPIRIT) [29] and the Brazilian Regulatory Guidelines and Norms for Research Involving Human Subjects of the National Health Council of the Ministry of Health, December 2012, were followed. The study was approved by the Ethics Committee for Research with Human of Santa Casa de Misericórdia (process number 178/2012) and was registered at ClinicalTrials.gov (NCT02409160). All volunteers provided informed consent and were notified of their ability to leave at any time without any cost. The study design is shown in Figure 1.

Obesity was classified according to the WHO established criteria that define BMI as body weight in kilograms divided by the square of the patient’s height in meters (kg/m^2^). For this study, obesity was stratified into five distinct classes based on BMI: class I (30 to 34.9 kg/m^2^), class II (35.0 to 39.9 kg/m^2^), class III (40.0 to 49.9 kg/m^2^) [2,30,31], class IV (50.0 to 59.9 kg/m^2^), and class V (≥60 kg/m^2^) [3,32].

### 2.2. Study Setting

This study was conducted at the Faculty of Medical Sciences of Santa Casa de São Paulo, Brazil between January and December of 2019. The doctors and surgeons of the hospital’s obesity surgery department performed all surgical procedures. Blood samples were collected at Hospital da Irmandade da Santa Casa de Misericórdia de São Paulo and the Immunology Laboratory of the Federal University of São Paulo, São José dos Campos.

### 2.3. Participants and Eligibility Criteria

The volunteers, clinically stable, were consecutively recruited from the Surgery Unit of Santa Casa de Misericórdia de São Paulo and selected in accordance with the eligibility criteria previously established by the study protocol. Our inclusion criteria allowed patients aged between 18 and 65 years with severe obesity level III (BMI ≥ 40 kg/m^2^) or with obesity level II (BMI ≥ 35 kg/m^2^) associated with comorbidities; with or without MetS; with a clinical indication for bariatric surgery according to the criteria of the Conselho Federal de Medicina (Brazil); a documented history of failure in conventional weight loss; and the ability to understand, agree, and sign the informed consent form. The exclusion criteria were as follows: a BMI of >55 kg/m^2^, an unrealistic postoperative target weight in conjunction with unrealistic expectations regarding surgical treatment, pregnancy, planned pregnancy within 2 years, breastfeeding, active cancer, clinical and/or mental instability, mental illnesses (schizophrenia or depression), epilepsy, and underlying disease genetics. In addition, patients with an absence of a safe-access gastrointestinal tract or abdominal cavity, who reported abuse of alcohol or drugs, and/or any medical conditions that contraindicated surgery were also excluded.

We conducted a sample size calculation based on a previous study by Rafey et al., who identified plasma levels of adiponectin in patients with severe obesity undergoing BS. Considering a margin of error of 0.05 and a significance level of 95%, we determined that 25 patients were required to detect an effect size of 0.95 [33].

### 2.4. Recruitment and Randomization

Based on the inclusion and exclusion criteria, the female patients who were in urgent need of surgery made up the bariatric surgery group (BSG), whereas the other patients, particularly those who did not meet the inclusion criteria and/or had no urgent need for surgery composed the control group (CG), that is, patients in the CG, did not undergo the surgical procedure for a period of 6 months. It is important to mention that the investigators who participated in the clinical trial interpretation were blinded to the study group’s composition. The patients were examined preoperatively and again at 6 months following each surgical procedure. The CG participants were assessed at the baseline when randomization took place and again at 6 months following the first assessment. All patients were evaluated by the same researchers and were also instructed to maintain their daily routines, especially regarding eating habits and physical activity. Any change in routine was required to be communicated to the researchers.

### 2.5. Protocol for Clinical and Surgical Assessment

Data regarding anthropometrics, demographics, biochemicals, the use of medication, and also clinical and surgical history were obtained from all participants. All evaluations were performed both before and after 6 months of the surgical procedure for the BSG or after the first evaluation for the CG by a consistent team, which included physicians, physical therapists, and researchers.

Clinical evaluation of the patients was performed at the Bariatric Surgery Outpatient Clinic of Santa Casa de Misericórdia de São Paulo. To determine weight and height, each participant was instructed to empty the bladder and wear light clothes without shoes, and it a digital electronic anthropometric scale was used (model 200/5, Welmy Indústria e Comércio Ltd.a., São Paulo, Brazil). BMI was calculated through the following equation: weight (kg) divided by height squared (m^2^), following the WHO classification [2,34].

Clinical information regarding signs and symptoms including the presence of cough, sputum, dyspnea, fatigue, associated diseases and possible exacerbations, drug use, and comorbidities were collected from the medical records of patients at Hospital da Brotherhood of the Santa Casa de Misericórdia de São Paulo.

### 2.6. Blood Biochemical Analysis

Fasting blood samples (5 mL) were collected, preoperatively, in vacuum tubes (Vacuette do Brasil Ltd.a, Campinas, Brazil) containing EDTA anticoagulant (Merck KGaA, Darmstadt, Germany) via cubital venipuncture for plasma preparation. Circulating levels of total cholesterol, HDL, LDL, triglycerides, and glucose were assessed using commercial kits (Gold Análise Diagnostica Ltd., MG, Brazil) for the SpectraMax i3 analysis system (Molecular Devices, Sunnyvale, CA, USA). The inflammatory markers leptin and adiponectin were assessed using commercial ELISA kits (R&D Systems, Minneapolis, MN, USA, and BioLegend, Sellex Inc., Washington, DC, USA), following manufacturer recommendations.

### 2.7. Surgical Procedures

The RYGB technique is one of the most commonly used surgical procedure for obesity. This procedure combines restrictive aspect because of small gastric pouch creation and metabolic effects due to the total bypass of food from the duodenum and proximal jejunum with high incretinic response. Surgical procedures were performed at the Department of Bariatric Surgery of the Irmandade da Santa Casa de Misericórdia de São Paulo linked to the Faculty of Medical Sciences of Santa Casa de São Paulo by five surgeons who used a standard open-RYGB technique [35,36]. The clinical success of this procedure can be partly attributed to its alteration of the secretion of hormones that influence glucose regulation and the patient’s perception of hunger and satiety. All patients underwent identical anesthetic and surgical protocols.

### 2.8. Statistical Analysis

In order to avoid possible trends in the results, initially, an identification of outlier values was performed. After that, the Shapiro–Wilk and D’Agostino and Pearson tests were used to verify the proximity of the data with a normal curve, followed by the variance homogeneity analysis, which was assessed using Levene’s test.

Based on it, parametric data are presented as means and standard deviations, and using Student’s t-test the occurrence of significant differences between the means of the values of these variables was verified. In addition, the one-way ANOVA with Dunn’s post-test was used to determine differences between control and experimental variables. Pearson’s coefficient correlation test was also performed.

Concerning the non-parametric data, their values were presented as medians and interquartile ranges, and by using the Wilcoxon test it was possible to assess the occurrence of significant differences between these variables. Additionally, the Friedman test was performed to determine differences between the control and experimental variables. Spearman´s coefficient correlation test was also used.

Categorical data are presented as percentages and absolute numbers. All these analyses were performed using the Statistical Package for Social Sciences (SPSS, 21.0, IBM Corp., Armonk, NY, USA), and the significance level was set at 5% (*p* < 0.05) for all statistical tests.

## 3. Results

First of all, it is important to mention that there was a predominance of white women (88%) followed by black or other (12%) women, with a mean age of 42.1 years (±12.7) for the CG and 42.5 (±9.8) for the BSG. In addition, it is worth mentioning that 42% of volunteers were in menopause, and most participants were married (52%), followed by single women. (40%) and widows (10%); 88% reported having never smoked, whereas 12% smoked in the past, while 94% were not alcoholic and 6% reported regular alcohol drinking.

Table 1 shows the anthropometric and biochemical characteristics of the patients involved in this study, divided into CG (*n* = 11) and BSG with or without MetS (*n* = 50) for the first and second assessments as well as before and after bariatric surgery. The values obtained in the post-surgery BSG showed significant reductions compared to the preoperative values.

Table 2 shows the anthropometric characteristics of the 50 patients who received bariatric surgery subdivided into the severe obesity bariatric surgery group (SOBSG) and the metabolic bariatric surgery group (METSBSG). Both groups showed significant reductions in weight and BMI following surgery when compared to pre-surgery values, with no difference being observed between the two groups at the time points evaluated.

The values from the plasma analysis of glycemic levels and lipid profiles (total cholesterol and fractions (LDL and HDL) and triglycerides) of patients divided into SOBSG and METSBSG can be seen in Table 3. In both groups, the values found after bariatric surgery were significantly different from the preoperative values. However, in the intergroup analysis, while pre-surgery triglyceride values were higher in the SOBSG than in the METSBSG, the values found in the post-surgery period were not different between the volunteer groups.

The circulating levels in blood plasma of the inflammatory markers adiponectin and leptin and their ratio in patients undergoing bariatric surgery subdivided into SOBSG and METSBSG are shown in Table 4. While circulating adiponectin levels increased significantly, leptin levels were significantly reduced after bariatric surgery when compared to the pre-surgery values. These data showed a more significant relationship between plasma levels of adiponectin and leptin being observed post-surgery than pre-surgery. No differences were observed between the two groups.

## 4. Discussion

Our results showed that in female patients with severe obesity with or without MetS, weight loss induced by bariatric surgery with RYGB improved biochemical and systemic inflammatory parameters, particularly the adiponectin/leptin ratio. In line with the existing literature, our findings from pre- and post- surgical evaluations showed a relationship between MetS and severe initial obesity resulting from increases in leptin levels and reductions in adipose levels. In one randomized study, patients undergoing serial gastroplasty with unspecified forms of T2DM were selected for IL-6, IL-10, and IL-10. The authors observed significant improvements in leptin (*p* ≤ 001) and CRP (*p* = 0.003) values at 1 and 6 months following bariatric surgery, as well as lower IL-6 levels at 6 months (*p* = 0.001). However, in a different way to the present method used here, the adiponectin levels between patient groups did not show a significant difference [37].

It is known that leptin and adiponectin present opposite effects on insulin resistance and subclinical inflammation, whilst leptin can upregulate pro-inflammatory cytokines, such as IL-6 and TNF-α, both molecules being closely related to insulin resistance and T2DM, while adiponectin, through its capacity to downregulate the expression and release of several pro-inflammatory immune mediators, shows anti-inflammatory properties. Thus, interactions between angiotensin II and adiponectin/leptin imbalances may be important mediators of the T2DM and cardiovascular disease risks associated with abdominal obesity [38]. Considering this information, the higher systemic levels of adiponectin and lower leptin observed in this study may have been associated with a decrease in subclinical inflammation in participants with severe obesity and MetS.

Confirming the hypothesis of reduced inflammation for our study population, we observed an improvement in the adiponectin/leptin ratio, a biomarker for inflammation in adipose tissue [39]. This ratio may be used to estimate the cardiometabolic risk associated with obesity and MetS, allowing for a broad identification of at-risk individuals [17]. Fruhbeck et al. showed that a reduced adiponectin/leptin ratio was associated with higher levels of the inflammation markers CRP and serum amyloid A (SAA), demonstrating that in severe obesity and MetS, adipose tissue dysfunction characterized by the adiponectin/leptin ratio leads to an increase in pro-inflammatory factors as potential mediators in their etiopathogenesis [39].

In addition to being good markers of adipose tissue-associated inflammation, both leptin and adiponectin are involved in the regulation of lipolysis, and thus reductions in adiponectin/leptin ratios may also reflect changes in this process, further metabolically contributing to obesity [40]. Therefore, this emerging and promising biomarker has shown a more prominent correlation not only with insulin resistance than the evaluation of leptin and adiponectin alone has, but also demonstrates a remarkable reduction compared to the higher number of metabolic risk factors [41]. In this context, the findings of this study suggest that an increase in the adiponectin/leptin ratio could be related to the reduction in glucose levels observed after BS.

Similarly, adiponectin/leptin ratio values also showed a high negative correlation with several low-grade chronic inflammation markers; they have emerged as an important predictive marker of cardiometabolic risk associated with obesity and MetS. Hence, elevations in this proportion have been related to a reduced risk of the development of atherosclerosis and some types of cancer [41]. In recent years, it has been demonstrated that adipose tissue is an extremely active endocrine organ that produces diverse biologically active adipokines such as adiponectin, leptin, TNF-α, and IL-6, that participate in several physiological processes [42], which include the pivotal role in the pathophysiological association between higher adiposity and cardiometabolic alterations [43,44]. In order to verify whether or not the adiponectin/leptin ratio could contribute to the pathophysiology of increased oxidative stress and systemic inflammation in patients with MetS, Fruhbeck et al. assessed the systemic levels of adiponectin, leptin, and other inflammatory and oxidative stress markers in Caucasian individuals (*n* = 140; men = 74 and women = 66; aged between 28 to 82 years; 60 participants had MetS and 80 did not have MetS). In an interesting way, these authors reported not only a significant reduction in circulating adiponectin levels but also that the adiponectin/leptin ratio was dramatically decreased in MetS patients [41]. According to their study, individuals with MetS show systemic elevations in both oxidative stress, which can be verified in terms of thiobarbituric acid reactive substances (TBARS) levels, and inflammatory markers, such as osteopontin, CRP, and SAA. In this respect, the authors reported the occurrence of negative correlations between circulating adiponectin concentrations with TBARS, an oxidative stress marker, as well as with CRP, an inflammatory marker. Based on these pieces of information, these authors concluded that MetS is closely associated with both increased oxidative stress and chronic pro-inflammatory status and also that a low adiponectin/leptin ratio, which can be associated with dysfunctional adipose tissue, is an important player in the increased inflammation and oxidative stress found in MetS [44]. The data of our study corroborate the study from Fruhbeck et al., in which it was possible to observe in a serial manner increases in the adiponectin/leptin ratio as well as a decrease in triglyceride levels, total cholesterol, HDL, and LDL following surgery using the RYGB technique [39].

Notably, the results of a systematic review conducted by Lopez-Jaramillo et al. reinforce the role of the adiponectin/leptin ratio. In fact, the authors showed that in patients who presented with severe coronary artery disease, the lower circulating adiponectin levels and higher leptin levels were exclusively related to abdominal obesity. Moreover, an imbalance in the adiponectin/leptin ratio has also been related to a higher waist circumference, lower vascular response to acetylcholine, and elevation in vasoconstriction due to angiotensin II [36]. In the study of Unamuno et al., the authors observed a significant improvement in the anthropometric and biochemical variables (*p* < 0.0001) of 22 patients with severe and T2D obesity undergoing RYBG. The adiponectin/leptin ratio also showed a significant increase (*p* <0.001) compared to the pre- and post-surgery values; however, the behavior of this relationship was no different between patients with and without T2D [45]. These findings corroborate those of our study regarding the adiponectin/leptin ratio that showed improvements after RYGB-induced weight loss in patients with severe obesity and T2D.

In a study involving 292 individuals considered according to the BMI to be lean (83, 28%) and obese (209, 72%), the authors investigated the reliability of the adiponectin/leptin ratio as a marker of metabolic dysfunction. A significant negative correlation was observed between the adiponectin/leptin ratio and serum amyloid A (SAA), an important marker of adipose tissue dysfunction. Fruhbeck et al. with these results propose that the adiponectin/leptin ratio is an excellent marker of adipose tissue dysfunction inducing increased cardiometabolic risk [17].

Therefore, in line with the results of these recent studies, it is believed that the adiponectin/leptin ratio may be an important inflammatory marker that can be used to investigate comorbidities in patients with severe obesity and MetS before and after bariatric surgery.

In a recent literature review published by Iron et al., the current scientific knowledge regarding obesity, metabolic syndrome, and the effects of weight loss induced by the practice of physical activities, diets, pharmacological treatments, and/or surgical procedures was increased, since the authors concluded that a reduction in BMI, regardless of the method, improves glycemic control, reduces the concentration of pro-inflammatory proteins and increases that of anti-inflammatory markers, consequently significantly decreasing cardiovascular risk factors [46].

According to the existing literature, bariatric and metabolic surgery have been effective at maintaining weight loss and metabolic improvement in patients with a BMI of > 40 kg/m^2^ or a BMI of > 35 kg/m^2^ in the presence of comorbidities, especially for those with a history of failure in previous conservative therapeutic approaches for weight reduction [47]. Similar to the findings of our study, previous authors have demonstrated excellent health outcomes for individuals with obesity or MetS undergoing RYGB including a remission of comorbidities, reduction in systemic inflammation, and significant reduction in high CRP levels [48,49].

One of the limitations of this study was the fact that it involved only female patients. This fact is justified by the greater demand for bariatric surgery in our hospital from women with severe obesity. The postoperative follow-up period of just six months could be considered a limitation. However, a study showing the behavior of the adiponectin/leptin ratio in the medium and long term after surgical procedures would be very interesting. As a strong point of our study, we highlight the results that are in line with the literature, reinforcing the importance of considering the adiponectin/leptin ratio in obese patients with and without metabolic syndrome.

## 5. Conclusions

Taken together, our results allow us to conclude that the weight loss promoted by BS in female patients with severe obesity presenting or not presenting with MetS could remarkably improve anthropometric, biochemical, and systemic inflammatory variables, especially the ratio of adiponectin to leptin. Furthermore, our data, in conjunction with previous reports in the literature, can reinforce to the scientific community the importance of the adiponectin to leptin ratio as an emerging inflammatory biomarker, particularly for cardiometabolic risk. Another detail to be highlighted was the impact caused by RYGB-induced weight loss on the inflammatory profile of severely obese patients with and without MetS.

## Figures and Tables

**Table 1:** Anthropometrics, demographics, and clinical characteristics of the patients enrolled in the study.

Parameters	Control Group (*n* = 11)		*p*	Bariatric Surgery (BS) Group (*n* = 50)		*p*
	Evaluation 1	Evaluation 2		Pre-Surgery	Post-Surgery	
Weight	108 ± 17.12	112.64 ± 17.75	0.0083	123 ± 20.35	85.5 ± 15.29	<0.0001
BMI	45.73 ± 5.45	46.35 ± 5.06	0.0102	47 ± 6.25	33 ± 5.45	<0.0001
Triglycerides	120 ± 42.22	124.82 ± 34.31	0.9226	148 ± 57.05	90 ± 19.91	<0.0001
Total Cholesterol	196 ± 23.34	194.00 ± 21.07	0.9341	196 ± 33.25	110 ± 21.74	<0.0001
LDL cholesterol	123 ± 35.9	121.18 ± 20.77	0.3864	131 ± 30.24	97 ± 20.13	<0.0001
HDL cholesterol	42 ± 6.94	44.18 ± 5.75	0.4381	45.5 ± 13.15	53 ± 10.65	0.0054
Glycemia	91 ± 8.06	104.27 ± 6.62	0.0165	103 ± 13.05	86 ± 8.27	<0.0001

Note: BMI, body mass index; HDL, high-density lipoprotein; LDL, low-density lipoprotein; *p*, statistical significance value.

**Table 2:** Anthropometric characteristics of the patients enrolled in the study divided into the severe obesity bariatric surgery group and the metabolic syndrome bariatric surgery group.

Parameters	Severe Obesity Bariatric Surgery Group (*n* = 29)		*p*	Bariatric Surgery Group Metabolic Syndrome (*n* = 21)		*p*	*p*	
	Pre-BS	Post-BS		Pre-BS	Post-BS		Pre-BS	Post-BS
Weight	119.81 ± 11.8	83.72 ± 11.6	<0.0001	127.07 ± 21.4	88.77 ± 15.8	<0.0001	0.6192	0.5468
BMI	44.66 ± 5	31 ± 4.7	<0.0001	48 ± 6.2	34 ± 5.4	<0.0001	0.3285	0.4534

Note: BS, bariatric surgery; BMI, body mass index; *p*, statistical significance value.

**Table 3:** Blood biochemistry of the patients involved in the study, divided into the severe obesity bariatric surgery group and the metabolic syndrome bariatric surgery group.

Parameters	Severe Obesity Bariatric Surgery Group (*n* = 29)		*p*	Bariatric Surgery Group Metabolic Syndrome (*n* = 21)		*p*	*p*	
	Pre-BS	Post-BS		Pre-BS	Post-BS		Pre-BS	Post-BS
Glucose	107.75 ± 14.4	83 ± 9.12	<0.0001	102.92 ± 12.3	86.53 ± 7.9	0.0007	0.6378	0.3656
TGC	184.36 ± 67.3	95.27 ± 29.2	<0.0001	145.51 ± 49.3	89.84 ± 16.4	0.0006	0.0384	0.4132
Total Cholesterol	208.72 ± 45.1	132.27 ± 24.3	<0.0001	195.23 ± 28.6	118.58 ± 19.3	0.0002	0.5294	0.2939
HDL	41.45 ± 13.0	54.97 ± 5.7	0.0075	48.09 ± 12.6	52.32 ± 11.2	0.0284	0.3418	0.6037
LDL	129.27 ± 29.9	95.63 ± 13.1	<0.0001	134.28 ± 30.2	97.66 ± 21.2	0.0012	0.6303	0.7152

Note: BS, bariatric surgery; TGC, triglycerides; HDL, high-density lipoprotein; LDL, low-density lipoprotein; *p*, value of statistical significance.

**Table 4:** Adiponectin and leptin values and the adiponectin to leptin ratio of the patients involved in the study, divided into the severe obesity bariatric surgery group and the metabolic syndrome bariatric surgery group.

Parameters	Severe Obesity Bariatric Surgery Group (*n* = 29)		*p*	Bariatric Surgery Group Metabolic Syndrome (*n* = 21)		*p*	*p*	
	Pre-BS	Post-BS		Pre-BS	Post-BS		Pre-BS	Post-BS
Adip	0.33 ± 0.3	1.23 ± 0.4	<0.0001	0.42 ± 0.3	1.28 ± 0.5	0.0018	0.345	0.797
Lep	1.83 ± 0.5	0.32 ± 0.3	<0.0001	2.01 ± 0.5	0.52 ± 0.4	0.0015	0.388	0.124
Adip/Lep	0.16 [0.04–0.041]	2.70 [1.52–5.4]	0.0053	0.1 [0.03–0.35]	10.2 [2.31–23.57]	0.0084	0.548	0.192

Note: BS, bariatric surgery; Adip, adiponectin; Leptin, leptin; Adip/Lep, ratio of adiponectin to leptin; *p*, value of statistical significance.

## Data Availability

The data generated in this study will be available to the scientific community upon request.
